# Predicting post-surgical functional status in high-grade glioma with resting state fMRI and machine learning

**DOI:** 10.1007/s11060-024-04715-1

**Published:** 2024-05-24

**Authors:** Patrick H. Luckett, Michael O. Olufawo, Ki Yun Park, Bidhan Lamichhane, Donna Dierker, Gabriel Trevino Verastegui, John J. Lee, Peter Yang, Albert Kim, Omar H. Butt, Milan G. Chheda, Abraham Z. Snyder, Joshua S. Shimony, Eric C. Leuthardt

**Affiliations:** 1 Department of Neurological Surgery, Washington University School of Medicine, St. Louis, MO USA; 2 Center for Health Sciences, Oklahoma State University, Tulsa, OK USA; 3 Mallinckrodt Institute of Radiology, Washington University School of Medicine, St. Louis, MO USA; 4 Department of Neurology, Washington University School of Medicine, St. Louis, MO USA; 5 Department of Biomedical Engineering, Washington University in Saint Louis, St. Louis, MO USA; 6 Department of Neuroscience, Washington University School of Medicine, St. Louis, MO USA; 7 Department of Mechanical Engineering and Materials Science, Washington University in Saint Louis, St. Louis, MO USA; 8 Center for Innovation in Neuroscience and Technology, Washington University School of Medicine, St. Louis, MO USA; 9 Brain Laser Center, Washington University School of Medicine, St. Louis, MO USA; 10 National Center for Adaptive Neurotechnologies, Albany, NY USA

**Keywords:** Brain tumor, High-grade glioma, Functional MRI, Machine learning

## Abstract

**Purpose:**

High-grade glioma (HGG) is the most common and deadly malignant glioma of the central nervous system. The current standard of care includes surgical resection of the tumor, which can lead to functional and cognitive deficits. The aim of this study is to develop models capable of predicting functional outcomes in HGG patients before surgery, facilitating improved disease management and informed patient care.

**Methods:**

Adult HGG patients (*N* = 102) from the neurosurgery brain tumor service at Washington University Medical Center were retrospectively recruited. All patients completed structural neuroimaging and resting state functional MRI prior to surgery. Demographics, measures of resting state network connectivity (FC), tumor location, and tumor volume were used to train a random forest classifier to predict functional outcomes based on Karnofsky Performance Status (KPS < 70, KPS ≥ 70).

**Results:**

The models achieved a nested cross-validation accuracy of 94.1% and an AUC of 0.97 in classifying KPS. The strongest predictors identified by the model included FC between somatomotor, visual, auditory, and reward networks. Based on location, the relation of the tumor to dorsal attention, cingulo-opercular, and basal ganglia networks were strong predictors of KPS. Age was also a strong predictor. However, tumor volume was only a moderate predictor.

**Conclusion:**

The current work demonstrates the ability of machine learning to classify postoperative functional outcomes in HGG patients prior to surgery accurately. Our results suggest that both FC and the tumor’s location in relation to specific networks can serve as reliable predictors of functional outcomes, leading to personalized therapeutic approaches tailored to individual patients.

**Supplementary Information:**

The online version contains supplementary material available at 10.1007/s11060-024-04715-1.

## Introduction

High-grade gliomas (HGG) account for 60–70% of new cases of brain tumors [1]. The efficacy of therapeutic interventions has been limited by various factors, such as genetic heterogeneity, accelerated cellular proliferation, and treatment-resistant cells [1–4]. Consequently, the median survival rate for patients afflicted with HGGs remains low, with a median survival of merely 14 months. Standard clinical practice typically entails gross total resection of the tumor, followed by radiation and adjuvant chemoradiotherapy [2]. However, surgical resection of the tumor carries the risk of causing or exacerbating functional impairment, a significant concern for the clinician, as postsurgical functional preservation (e.g., quality of life) is known to correlate with overall patient outcomes [5–9]. To that end, the capability to forecast postsurgical functional outcomes from the initial diagnosis could prove advantageous in surgical planning and for better-informing patients of their likely treatment outcomes.

Structural (e.g., T1-weighted pre and post-contrast, T2-weighted, and diffusion tensor images (DTI)) and functional (e.g., blood oxygen level-dependent task or resting state fMRI) MRI data are routinely acquired in the pre-surgical setting. The former assesses tumor size and location, while the latter helps evaluate a planned resection’s functional and cognitive implications. Using the various MRI modalities as a guide, the surgeon faces the challenge of achieving maximal resection of infiltrative tissue while minimizing functional morbidity. These competing interests, long-term survival through maximum resection versus quality of life through functional preservation, significantly impact overall patient outcomes, and finding the optimal balance between the two is an ongoing challenge [5–8].

Task fMRI (T-fMRI) has traditionally been the conventional non-invasive approach for mapping functional brain networks before surgical intervention. However, T-fMRI has limitations that can constrain clinical utility [10]. For example, the need for patients to engage in specific cognitive or motor tasks during the scan can pose challenges and may even be impractical for certain individuals. Alternatively, resting state fMRI (RS-fMRI) allows researchers to examine the intrinsic functional connectivity of the brain and the associated interactions between different networks without the confounding effects of task performance [11]. Importantly, resting state functional mapping can be achieved under sedation and multiple networks can be mapped simultaneously, leading to a lower failure rate than T-fMRI [12]. Numerous studies have demonstrated the clinical utility of RS-fMRI in multiple disease categories, including brain tumors [7, 13–16].

Machine learning (ML) is a field of artificial intelligence that extracts patterns from data to build models with wide-ranging applications in medical research, including brain tumors [17]. Deep learning models have advanced image segmentation, enabling accurate classification of brain tissue categories (e.g., healthy, edema, necrotic/non-enhancing core, enhancing core) and brain network mapping [7, 18, 19]. MRI radiomic features have also proven valuable for predicting genetic features, identifying both pseudo and true tumor progression, and classifying transcriptome subtypes [20–22]. Given this information, we hypothesize that ML can extract and leverage pertinent features from RS-fMRI data to preoperatively predict HGG patients’ post-operative functional outcomes before surgical resection accurately.

This research aims to utilize ML and RS-fMRI to develop models capable of predicting functional outcomes in adult HGG patients. Specifically, random forest models were trained to classify HGG patients (*n* = 102) into positive functional outcomes (KPS ≥ 70) or negative functional outcomes (KPS < 70). Input to the models included age, measures of resting state functional network connectivity (FC), the tumor’s degree of overlap with each RSN, and tumor volume. Autoencoders were used for dimensionality reduction, and permutation feature importance identified the strongest predictors of functional outcomes. Our results indicate that these models can accurately predict postoperative functional outcomes at the time of initial diagnosis.

## Materials and methods

### Patients

One hundred two patients diagnosed with intracranial primary HGG were retrospectively recruited from the neurosurgery brain tumor service at Washington University Medical Center. All subjects were diagnosed with HGG on pathological examination of biopsy and resection-acquired brain samples at the Division of Neuropathology between May 2012 and September 2020. Definitive diagnosis was achieved based on histomorphological and immunohistochemical characteristics supportive of HGG using the appropriate WHO guidelines [23]. These findings include the presence of tumor cells with astrocytic-like appearance, microvascular proliferation, palisading necrosis, pleomorphic hyperchromatic nuclei, and frequent mitoses. Our cohort consists of 85 patients with the diagnosis of GBM IDH wildtype and 17 patients with GBM IDH mutant. Under the recent WHO 2021 guidelines, the 17 IDH mutant patients would be classified as Grade IV Astrocytoma, IDH mutant based on the advanced role of molecular diagnostics in CNS tumor taxonomy. Inclusion criteria included a new diagnosis of brain tumor (first occurrence), biopsy or surgical treatment, and the availability of pre-surgical structural and functional MRI. Exclusion criteria included patients younger than age 18 and patients who were lost to follow-up. The Washington University in St. Louis Institutional Review Board approved this study. The requirement for informed consent was waived by the IRB.

### Clinical characteristics

Noted clinical characteristics are summarized in Supplemental Tables 1 and include survival duration (the difference between the first clinical visit and the date of death), the extent of resection (gross total resection (GTR) or subtotal resection (STR)), O6-Methylguanine-DNA methyltransferase (MGMT) methylation status, epidermal growth factor receptor (EGFR) amplification status, telomerase reverse transcriptase (TERT) mutation status, isocitrate dehydrogenase 1 (IDH1) mutation status, and phosphatase and tensin homolog (PTEN) mutation status. Genetic data were assayed by the Foundation Medicine commercial laboratory (www.foundationmedicine.com). History (Hx) noted included alcohol use disorder, tobacco use, hypertension, hyperlipidemia, chronic kidney disease (CKD), cardiac issues, deep vein thrombosis or pulmonary embolism (DVT/PE), psychiatric disorders, visual deficits, stroke, weakness, and/or migraine/tension/cluster headaches. Presentation symptoms (Pw) noted included weakness, visual changes, hydrocephalus, confusion, headache, memory impairment, seizures, obesity (BMI > 30), and diabetes.

For each patient, the Karnofsky Performance Score was acquired from the electronic medical record. An Oncologist or Radiation Oncologist evaluated each KPS at the first (post-operative, pre-radiotherapy, or pre-chemotherapy) clinical visit. The methodology for the KPS followed standard research and clinical guidelines from the neuro-oncology literature [24, 25].

### MRI acquisition

All neuroimaging was performed on a Siemens Trio or Skyra 3T MRI scanner. Structural images included T1-weighted (T1w) magnetization prepared rapid acquisition gradient echo (MPRAGE: TE = 2.53ms, TR = 1900ms, TI = 900ms, 256 × 256 acquisition matrix, 0.976 × 0.976 × 1 mm voxels), fluid attenuated inversion recovery (FLAIR: 2D, slice thickness 5 mm, gap 1 mm, 256 × 256 matrix 0.9 × 0.9 mm pixel size, TE = 129ms, TR = 8500ms, TI = 2440ms, flip angle 130), and T2-weighted (T2w) fast spin-echo (FSE: TE = 93ms, TR = 5600ms, 256 × 256 acquisition matrix, 1.093 × 1.093 × 2 mm voxels). RS-fMRI was acquired using a blood oxygenation level-dependent (BOLD) sensitive echo planar imaging sequence (voxel size 3mm^3^ isotropic; echo time = 27ms; repetition time = 2.2–2.9 s; field of view = 256 mm; flip angle = 90). Details on MRI processing and automated tumor segmentation can be found in Supplemental material Sect. 1.1.

### RS-fMRI measures

Our analysis included two measures of RS-fMRI, connectivity and spatial overlap of the tumor with each RSN. The networks include dorsal somatomotor (SMD), inferior somatomotor (SMI), cinguloopercular (CON), auditory (AUD), default mode (DMN), parietal memory (PMN), visual (VIS), frontoparietal (FPN), salience (SAL), ventral attention (VAN), dorsal attention (DAN), medial temporal (MET), reward (REW), thalamus (THA), and basal ganglia (BGA). Distance correlation [26] was used to calculate network similarity, resulting in 120 measures. For spatial features, the dot product between the tumor segmentation maps and publicly available RSN probability maps [27] was calculated for each network described above. Further details for network similarity and spatial overlap features are described in Supplemental material Sect. 1.2.

### Machine learning and statistical analysis

Analyses were performed in MATLAB R2022b. Functional status was classified utilizing Random Forest models, an ensemble methodology comprising multiple decision trees [28]. Before training, dimensionality reduction via an autoencoder with a single hidden layer was used to reduce the FC feature space to 11 components labeled as FC1, FC2,…FC11. Model inputs included age, the encoded FC features, the spatial tumor features, and tumor volume. The model was trained to classify patients into two groups based on their Karnofsky Performance Status (KPS) score: KPS < 70 (negative functional outcome) and KPS ≥ 70 (positive functional outcome). All models were trained with 10-fold nested cross-validation (CV). Weighted classification was used during training to correct for class imbalance.

**Fig. 1:**
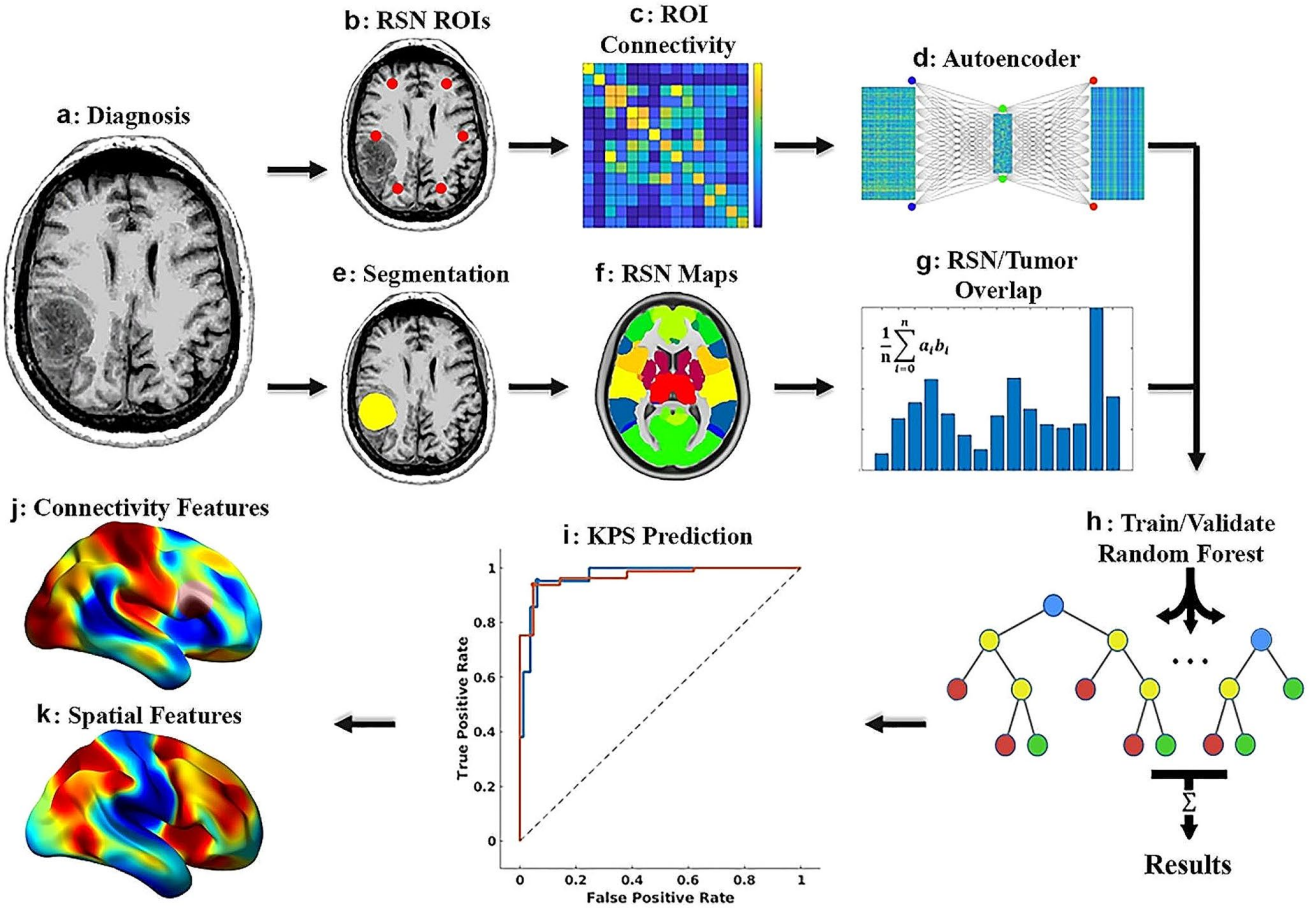
Functional outcome prediction pipeline. (**a**) Resting-state functional MRI (RS-fMRI) is collected at the time of diagnosis. Connectivity between network ROIs is calculated and passed through an autoencoder for dimensionality reduction (**b**–**d**). The tumor is segmented (**e**), and the segmentation is used to calculate the degree of overlap between the tumor and each RSN (**e**–**g**). The connectivity and spatial features are used to train a random forest to classify patients based on postoperative functional outcome status (KPS < 70, KPS ≥ 70, **h**–**i**). Feature selection is then used to identify the relevant RSN in the context of both connectivity and spatial location of the tumor (**j**–**k**)

The autoencoder was trained on 80% of the FC data, with 20% reserved for validation termination. Permutation feature importance [28, 29] was used to identify the strongest predictive model features. Average network feature weights were generated by averaging all within and between-network feature weights for each network. Then, voxel-wise feature maps were generated by multiplying the average feature weights with publicly available FC probability maps [27]. Further analysis details are described in Supplemental material Sect. 1.3. The functional outcomes prediction pipeline and methods are summarized in Fig. 1.

## Results

**Fig. 2:**
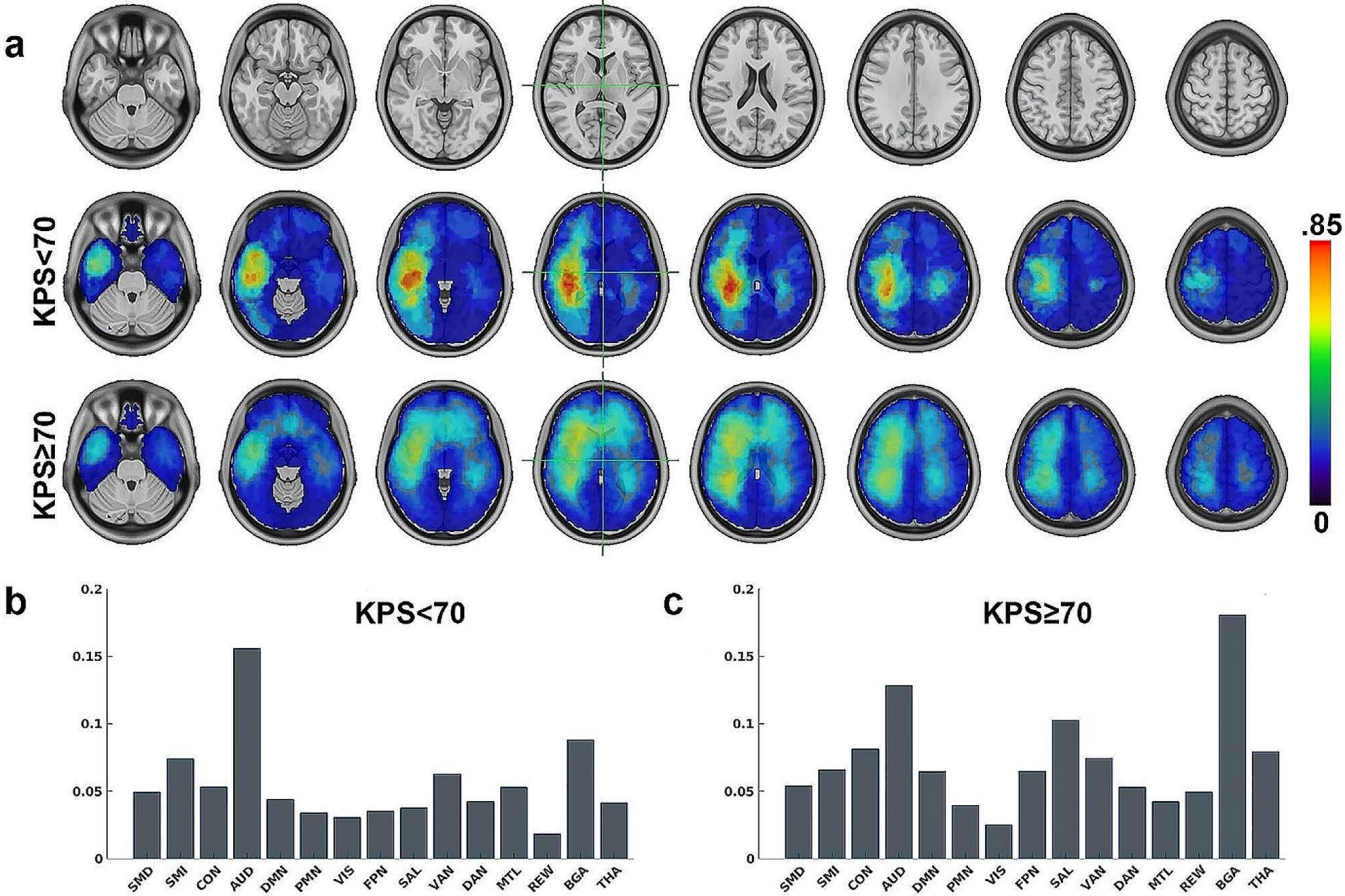
(**a**) Spatial tumor frequency maps for KPS < 70 and KPS ≥ 70. (**b**) Average degree of tumor overlap per RSNs for KPS < 70. (**c**) Average degree of tumor overlap per RSNs for KPS ≥ 70

Supplemental Table 1 describes patient characteristics, encompassing demographics, survival rates, classifications of surgical resections, identified genetic mutations, medical histories, and the symptoms at the time of diagnosis. Age at diagnosis and survival duration showed significant group differences (*p* <.02) between the positive and negative functional outcome groups, such that older age and lower survival duration were associated with poor functional outcomes. Similarly, a history of elevated incidence rates of hypertension and hyperlipidemia was associated with poorer functional outcomes (*p* <.01). Regarding presentation symptoms, higher rates of seizures were observed with poor functional outcomes (*p* =.02). The incident rate of presenting with headaches was approximately two-fold greater in the positive functional outcome group (*p* =.06). The median number of days between initial surgery and KPS was 25 days with median absolute deviation of 145 days. Figure 2A shows the regions exhibiting the most significant tumor presence, determined by averaging the tumor segmentation maps for all data and partitioned based on functional outcome group. Figure 2B shows the average degree of tumor overlap per RSN calculated by taking the dot product of the tumor frequency maps with published RSN probability maps [27] and normalized by the size of each RSN for KPS < 70. The most significant overlap was in AUD, BGA, and SMI networks. Similarly, Fig. 2C shows the overlap with KPS ≥ 70. Here, BGA was much more involved and AUD slightly less involved compared to KPS < 70.

**Fig. 3:**
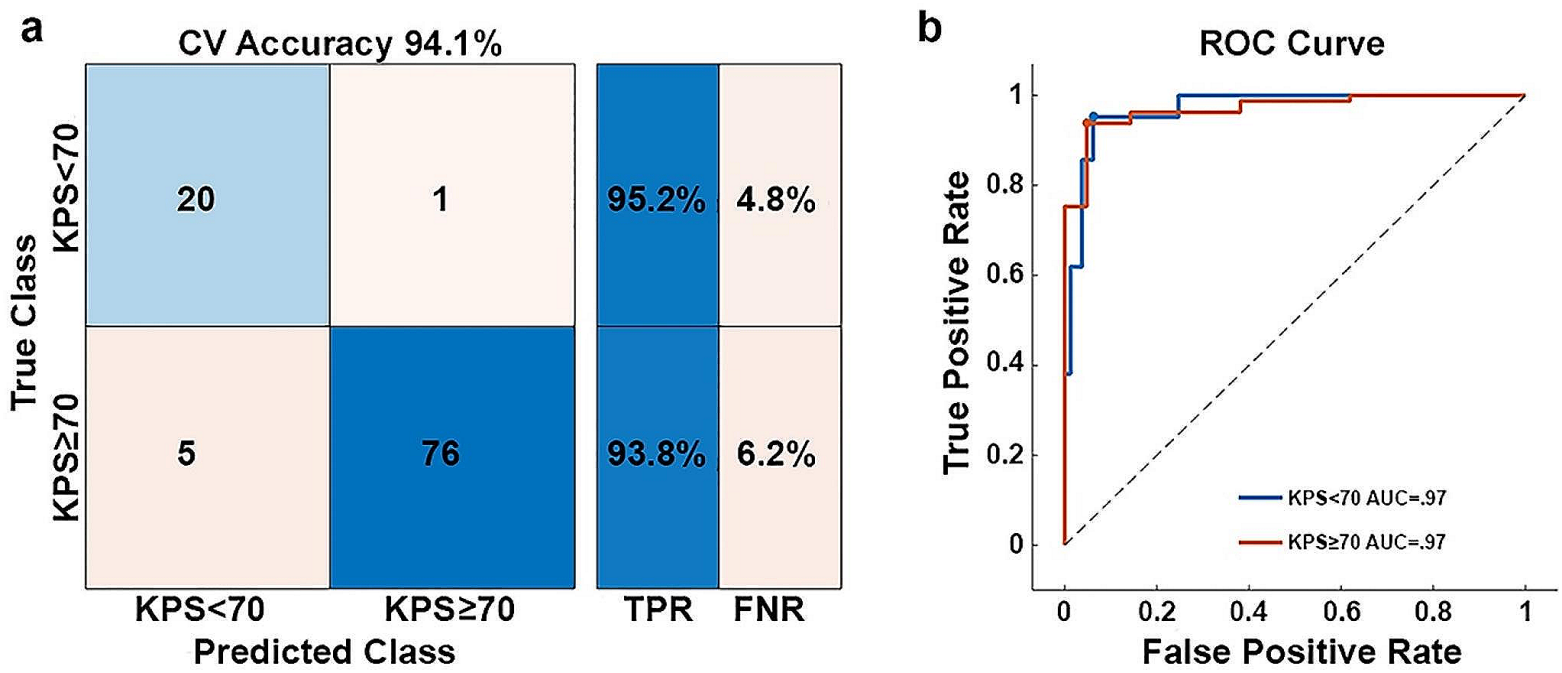
Model results. (**a**) The model achieved a nested cross-validation accuracy of 94.1%, with a minimum class specific accuracy of 93.8%. (**b**) The model achieved an AUC of 0.97

**Fig. 4:**
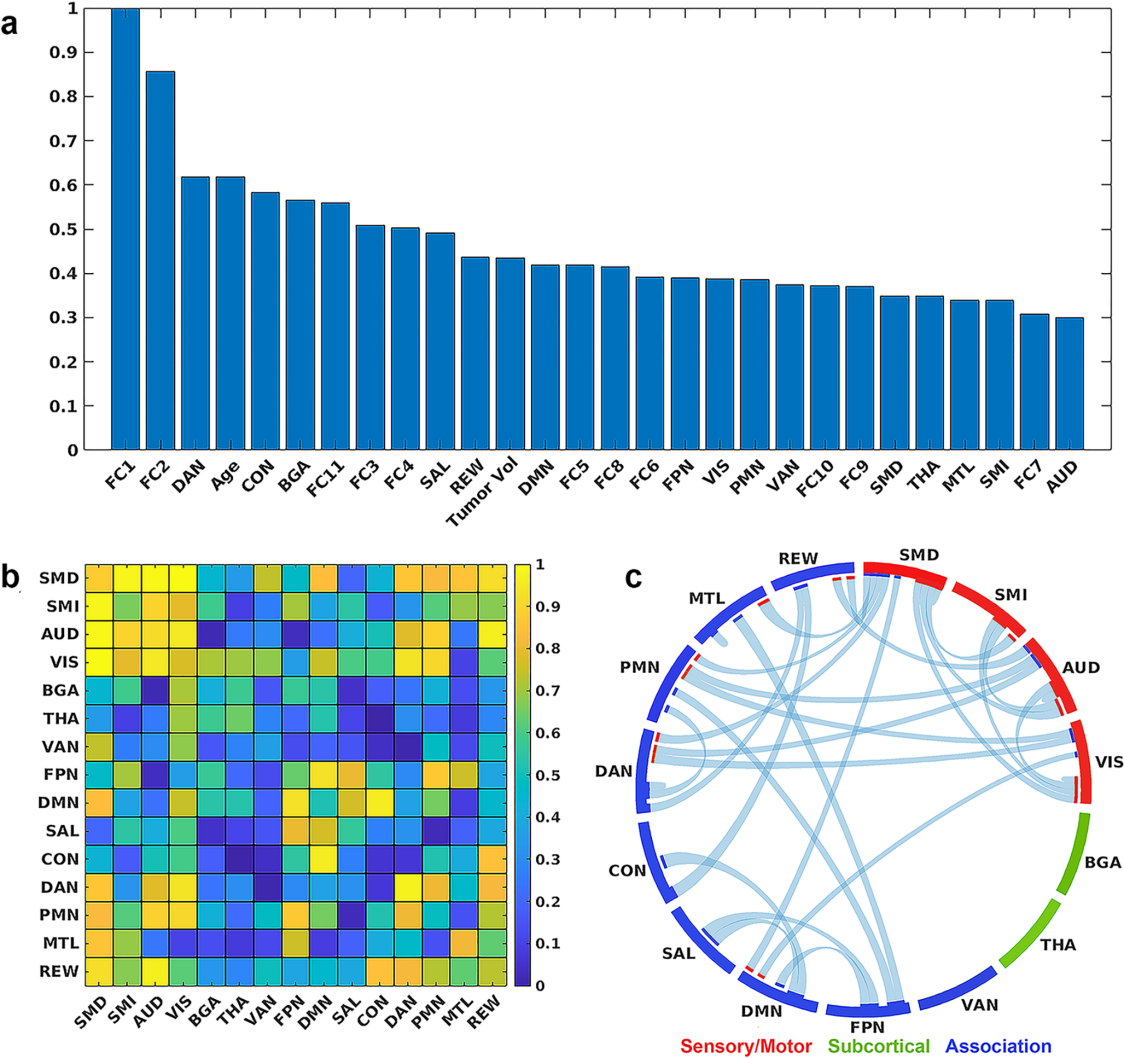
Top model features. (**a**) The strongest model predictors included encoded network connectivity features (FC1, FC2, and FC11). Based on location, tumors in dorsal attention, cingulo-opercular, and basal ganglia were strong predictors of KPS. Age was also a strong predictor. (**b**) Strongest resting state network connectivity features (rank) derived from permutation feature importance from FC1, FC2, and FC11 and grouped based on network functionality (sensory/motor, subcortical, and association). Overall, connectivity involving sensory and motor networks was the strongest predictor of KPS. (**c**) Top connectivity patterns (> 0.75) associated with the strongest resting state network correlation features in **b**

**Fig. 5:**
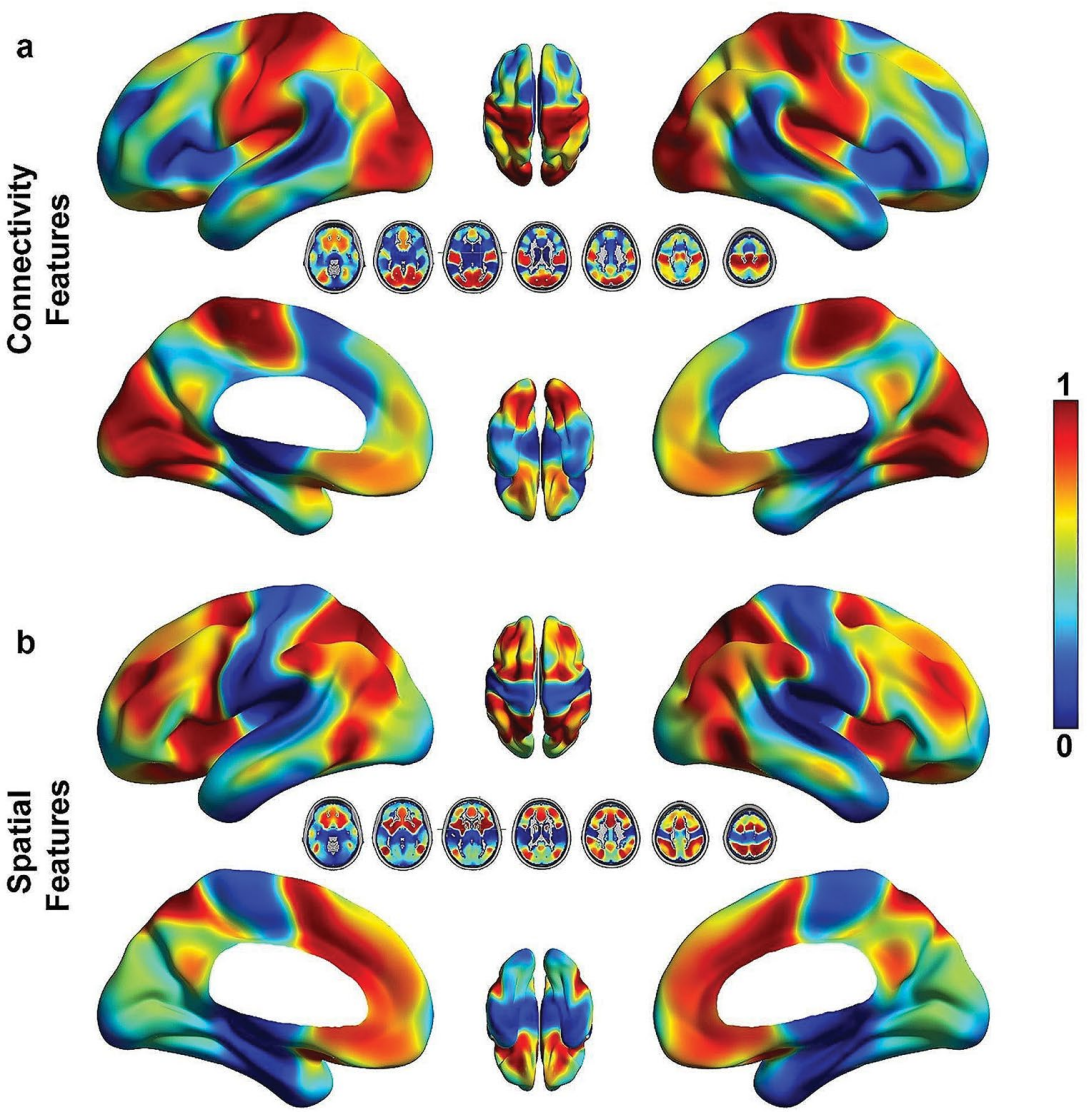
(**a**) Results of mapping the average network connectivity feature weights onto the published FC probability maps. The top regions include motor, auditory, and visual networks. (**b**) Results of mapping the spatial feature weights onto the published FC probability maps. These regions are associated with DAN, CON, BGA, and SAL networks

Figure 3 shows the model results. The final cross-validation accuracy in classifying KPS was 94.1% (true negative rate = 95.2%, true positive rate = 93.8%, Fig. 3A), with an AUC of 0.97 (Fig. 3B). Figure 4A describes the strongest predictive features identified by the model. Surprisingly, tumor volume was only a moderate predictor, but reveals the importance of tumor location for meaningful performance outcomes. However, age was among the top predictors. In the context of encoded network connectivity features, FC1, FC2, and FC11 were the strongest predictors. Permutation feature importance on the top encoded FC features (FC1, FC2, and FC11) showed primary involvement with networks associated with sensory and motor processes (SMD, SMI, VIS, and AUD, Fig. 4B and C). Some association networks (DMN, PMN, and DAN) showed moderate involvement, but these were primarily inter-RSN connectivity with sensory and motor networks. Connectivity involving VAN, THA, and BGA were not strong predictors. Based on the tumor’s location in reference to a given RSN, DAN, CON, and BGA were strong predictors of KPS (Fig. 4A). Figure 5A shows the results of mapping the rank of the average network *connectivity* feature weights (rank of means of columns in Fig. 4B) onto the published FC probability maps. The primary networks involved in the context of *connectivity* were motor, vision, auditory, and reward. Similarly, Fig. 5B shows the mapping of the features that were the strongest *spatial* predictors in reference to the tumor’s location (e.g., rank of spatial features in Fig. 4A: DAN, CON, BGA,…).

## Discussion

The study highlights the potential of machine learning in accurately classifying postsurgical functional outcomes among HGG patients. These findings indicate the feasibility of achieving classification accuracy exceeding 90% before surgical intervention, using basic demographics, tumor volume, and RS-fMRI measures. Further, these results were achieved despite substantial heterogeneity present in the patient data, encompassing diverse demographics, tumor characteristics, medical histories, and initial symptoms (Supplemental Table 1). This underscores the potential of integrating machine learning into clinical settings, particularly for informed decision-making in HGG patient management.

Predicting KPS in brain tumor patients prior to treatment can serve as a foundational tool for tailoring individualized treatment plans. First, it guides preoperative planning by helping surgeons choose the appropriate surgical approaches, balancing long-term survival with recovery and potential complications. Second, it informs the need for rehabilitation and support services, allowing healthcare teams to arrange personalized rehabilitation programs such as physical, occupational, or speech therapy for those with lower predicted KPS, while also coordinating supportive care services, including home care, mental health support, or community resources. Additionally, it helps create individualized monitoring and follow-up plans, determine the frequency of follow-up visits, and integrate quality of life assessments to monitor any changes in KPS. Furthermore, the predictive model can guide psychosocial support interventions, facilitating mental health interventions and family counseling services to manage the care responsibilities and emotional stress associated with HGG. Lastly, by providing patients with a clearer understanding of their likely outcomes, such models enhance the process of informed consent, allowing patients to make better decisions for themselves regarding their treatment options.

Ours is among the first to use RS-fMRI to predict functional outcomes in HGG directly. Historically, neuroimaging predictors of functional outcomes, survival, recurrence, and other clinically relevant determinants have been predominantly structural measures [14, 30, 31]. This preference for structural measures is partly due to the invasive nature of HGGs, which can be more readily visualized through structural imaging techniques. In contrast, task and resting state fMRI generally involve ROI-based analyses, which can have limited clinical utility. However, recent advancements at our and other institutions have leveraged deep learning and other emerging techniques to generate whole-brain RS-fMRI functional maps at both the individual and group levels, with group averages spanning thousands of individuals [13, 27, 32]. These techniques extend well beyond the simple mapping of language and motor functions. They account for many functional networks, offering a more nuanced evaluation of the cognitive, behavioral, and emotional impacts that a planned resection might have. This shift towards a functional approach provides a more comprehensive assessment of brain tumors, moving beyond the limitations of traditional structural anatomical location. Consequently, these advancements have opened new avenues for the research and clinical community to evaluate brain network connectivity in the context of brain tumor management. While further research is required, given the accuracy of our models and the context of predicting functional outcomes, functional MRI could potentially serve as a superior predictor compared to its structural counterparts for some outcome measures.

From a neurosurgical standpoint, the long-term survival of patients with HGGs is a balancing act between the extent of surgical resection of the lesion and functional preservation [5, 6, 33, 34]. While substantial research exists regarding survival prediction in relation to the extent of resection, investigations focusing on functional outcome predictions remain relatively limited and pose significant challenges [35]. Previous studies have identified factors such as tumor size, cranial nerve manipulation, major brain vessel manipulation, posterior fossa location, and eloquent cortex involvement as significant determinants of postoperative deterioration in KPS [36]. Our results indicate tumor volume was only a moderate predictor (Fig. 4A) of KPS, which might initially appear counterintuitive. However, this finding underscores the critical point that tumor location in relation to functional networks could have a more significant influence on functional outcomes than the size of the tumor alone.

Our findings show a distinct dichotomy between the anatomical location of the tumor and functional connectivity (FC) changes, specifically concerning their predictive ability for KPS. Interestingly, while the tumor *location* in reference to DAN and CON were strong predictors of KPS, changes involving SMD and VIS networks were the top *connectivity* predictors. This apparent dissociation may be due to several potential mechanisms. Prior works underscore the importance of control regions in executive control, including CON and DAN [37, 38]. Importantly, CON is closely associated with somatomotor networks [39, 40], a relationship further elucidated by the recent dual-system model of the primary motor cortex (M1) proposed by Gordon and colleagues [40]. This model presents M1 as comprising not only effector-specific regions for fine motor control but also the somato-cognitive action system (SCAN), which integrates controls, physiology, and body movement. Notably, the M1 SCAN region is functionally connected to CON. Viewed from this perspective, damage to CON could impact the M1 SCAN regions, potentially contributing to functional impairments observed in patients.

Additionally, numerous studies have reported lesion-induced disruptions of FC and associated these changes with behavioral deficits in clinical populations [41–43]. Although these studies have primarily focused on stroke, a condition with distinct etiology and lesion characteristics compared to HGG, the underlying principle linking lesion-induced disruption in the functional connectome to functional deficits remains pertinent. Moreover, focal brain lesions have been observed to exert far-reaching effects via damage to direct/indirect white matter structural connections, subsequently disrupting FC [44]. Other complications may also play a role in functional outcomes. For example, presenting with seizures was a significant indicator of poor functional outcomes (Supplemental Table 1). Seizures are common among HGG patients, and regardless of the cause, severe seizures are known to damage brain cells, possibly confounding the effects of HGGs on functional status [45, 46]. A history of hypertension was also strongly associated with poor functional outcomes. This is no surprise, as lifestyle factors, encompassing dietary habits, physical activity, stress management, and substance use, significantly contribute to the onset and progression of various diseases. Taken together, multiple parallel processes could potentially be responsible for FC disruption and associated functional impairment: damage to gray matter regions with specific functional network affiliations, the disruption of direct/indirect connections mediated by white matter damage, and overall health. While further research is warranted, specifically studies involving RSNs and white matter tractography, these results suggest the underlying mechanisms governing functional outcomes involve highly complex interactions between RSNs and their communication pathways.

Several limitations exist in the current work. Primarily, the data procured for this study were sourced from a single institution. In addition, the relatively small size of our data set limited the degree of validation we were able to perform (e.g., hold-out validation). Future work will involve prospective validation at our and other institutions, including comparison with experienced clinicians. Future work should also consider the inclusion of additional variables. We intentionally withheld numerous variables that could be clinically relevant to functional outcome prediction (e.g., genetics, medical history, etc.). This allowed us to focus more specifically on the utility of RS-fMRI to predict outcomes. Still, the inclusion of additional relevant variables could likely improve performance and offer a more holistic predictive model. Multimodal neuroimaging studies could expand our understanding of the complex interplay described above. Specifically, the combination of RS-fMRI with white matter measures, such as DTI, could shed further light on the relationship between the tumor’s spatial location, its effect on connectivity, and how the combination of the two manifests in the context of HGG. Lastly, pre-surgical KPS was not available for this study, which restricts our ability to assess baseline functional status. Despite this, predicting post-surgical KPS remains beneficial. Identifying cases where KPS does not change post-surgery can be as informative as detecting declines or improvements. Future studies should aim to include both pre- and post-surgical assessments to further validate and refine these predictions. Future work should also examine the influence of individual variables on postoperative KPS, including analyses that can separate the effects of variables, such as age and hypertension, from their potential relationship with preoperative KPS.

### Conclusion

This research demonstrates how machine learning can accurately classify functional outcomes in HGG patients prior to surgical, chemical, or radiotherapy treatments. These results were achieved using only age, tumor location, RS-fMRI measures, and tumor size. By incorporating these models into clinical practice, we stand to enhance patient care, enabling personalized treatment plans that balance quality of life with survival. Such models can drive a more nuanced approach to HGG patient management, prioritizing both longevity and post-treatment quality of life.

### Electronic supplementary material

Below is the link to the electronic supplementary material.

Supplementary Material 1

Supplementary Material 2

## Data Availability

The data used in this study will be made available after approval from the appropriate study PIs (Eric Leuthardt, Joshua Shimony).
