# Supplementary material for: Predicting post-surgical functional status in high-grade glioma with resting state fMRI and machine learning
Source: J Neurooncol. 2024 May 24;169(1):175–85. doi: 10.1007/s11060-024-04715-1 (PMC11269343; doi:10.1007/s11060-024-04715-1)
Supplement: Supplementary file 1 — Supplementary Material 1 [file 11060_2024_4715_MOESM1_ESM.pdf]

Predicting Survival in Glioblastoma with Multimodal Neuroimaging and Machine Learning
Journal of Neuro-Oncology
Luckett et al.
Luckett.patrick@wustl.edu

1.1 MRI Processing

Structural data preprocessing was performed with FreeSurfer (http://surfer.nmr.mgh.harvard.edu). T1w and T2w images were visually inspected to ensure brain structures were free of blurring, ringing, striping, ghosting, etc., caused by head motion. Three raters (B.L., D.D., and G.V.) reviewed the segmentations with an experienced neuroradiologist (JS) to ensure data quality [1]. Preprocessing of fMRI data followed previously described methods, including compensation for slice-dependent time shifts, elimination of systematic odd-even slice intensities, and rigid body correction of head movement [2, 3]. The data were then resampled and registered in 3mm^3 atlas space using affine transformations to the T1w structural image. Additional processing steps encompassed the elimination of linear trends on a voxel-wise basis, application of temporal low-pass filtering to preserve frequencies below 0.1Hz, regression of nuisance waveforms, and spatial smoothing using 6mm full width at half maximum Gaussian blurring in all directions. Frame censoring was implemented using the DVARS measure as previously described [2].

Automated tumor segmentation was performed with a pre-trained convolutional neural network (CNN) architecture [4] using post-contrast T1w, T2w, and Fluid Attenuated Inversion Recovery (FLAIR) scans. The algorithm segments tissue into vasogenic edema, necrotic/non-enhancing core, and enhancing core. Tumor segmentations underwent visual inspection for quality assurance, and any segmentations deemed inadequate were excluded. A whole tumor mask was used for masking during the registration of the structural and functional images. The automated tumor segmentation maps were also used to create maps showing the relevant frequency of a voxel overlapping with the tumor. For each map, regions not contained in the segmentation were assigned a value of zero, regions segmented as edema were assigned a one, and the non-enhancing/enhancing core was assigned a value of two. Subsequently, the individual maps were aggregated to generate heat maps illustrating the extent to which the presence of a tumor influences each voxel within the atlas.

1.2 RS-fMRI Measures

Our analysis included two measures of RS-fMRI, connectivity and spatial overlap of the tumor with each RSN. Connectivity measures were calculated as follows. Regions of interest (ROI) derived from past studies [5] were used to generate similarity maps for 15 resting state networks (RSN). ROIs were generated by taking each network's top 200 voxels with the highest probabilities. The networks include dorsal somatomotor (SMD), inferior somatomotor (SMI), cinguloopercular (CON), auditory (AUD), default mode (DMN), parietal memory (PMN), visual (VIS), frontoparietal (FPN), salience (SAL), ventral attention (VAN), dorsal attention (DAN), medial temporal (MET), reward (REW), thalamus (THA), and basal ganglia (BGA). Network similarity was calculated by computing the distance correlation [6] between the network-specific ROIs, resulting in 120 within and between similarity measures. Between-network correlations were calculated by comparing all 200 voxels for the two given networks in a single calculation. Within-network connections were calculated by computing the distance correlation between each voxel and the other 199 voxels and averaging all measures.

Spatial features were calculated as follows. First, tumor segmentation maps were generated for each individual (see section 2.4, automated tumor segmentation). Then, we acquired publicly available RSN probability maps [5]. These maps were generated from >2000 subjects and reflect the probability of each voxel belonging to each of the 15 RSNs described above. Then, we took the dot product of the tumor segmentation maps with the RSN probability maps over voxels for which the argmax (maximum probability) of the probability maps corresponds to the network in question (e.g., if we are calculating the spatial overlap for SMD, we only consider voxels that would be classified as SMD based on the argmax of

the probabilities for all 15 networks). We then normalize the measures by the number of voxels in the given calculation to account for networks of different sizes.

1.3 Machine Learning and Statistical Analysis

Analyses were performed in MATLAB R2022b. Functional status was classified utilizing Random Forest models, an ensemble methodology comprising multiple decision trees [7]. Each of the trees is independently trained on a distinct, randomly selected subset of the data, and the collective output of the trees forms the basis of the final model predictions. Before training, dimensionality reduction via an autoencoder with a single hidden layer was used to reduce the FC feature space to 11 components labeled as FC1, FC2,…FC11. The model inputs included age, the encoded FC features, the spatial relationship of the tumor with respect to the RSNs, and tumor volume. The model was trained to classify patients into two groups based on their Karnofsky Performance Status (KPS) score: KPS<70 (indicating a negative functional outcome) and KPS≥70 (indicating a positive functional outcome). All models were trained with 10-fold nested cross-validation (CV) stratified based on functional outcome, age, and tumor volume. Each outer fold reserved approximately ten samples for external validation, and the remaining samples for the corresponding outer fold were used to train the model with 10-fold cross-validation. Weighted classification was used during training to correct for class imbalance. The autoencoder was trained on 80% of the FC data, with 20% reserved for validation termination.

Permutation feature importance [7, 8] was used to identify the strongest predictive features of functional outcomes. Permutation feature importance involves randomly permuting each feature in the original dataset and evaluating the model's performance after each permutation. Comparing the model’s accuracy with permuted features to the accuracy without permuted features allows the identification of features that significantly affect model performance and are, therefore, considered strong predictors. This iterative process was repeated 100 times for each model corresponding to a specific cross-validation fold. Results were computed using the validation data reserved for the respective fold and aggregated and averaged to obtain a single weight for each feature. This process was used to identify the strongest predictive features of functional outcomes and which functional networks were most associated with each encoded FC feature. Average network feature weights were generated by averaging all within and between-network feature weights for each network. Then, voxel-wise feature maps were generated by multiplying the average feature weights with publicly available FC probability maps [5].

1. Lamichhane B, Luckett PH, Dierker D, et al (2023) Structural Gray Matter Alterations in Glioblastoma and High Grade Glioma-A Potential Biomarker of Survival. Neuro-Oncology Adv vdad034

2. Power JD, Barnes KA, Snyder AZ, et al (2012) Spurious but systematic correlations in functional connectivity MRI networks arise from subject motion. Neuroimage. https://doi.org/10.1016/j.neuroimage.2011.10.018

3. Shulman GL, Pope DLW, Astafiev S V, et al (2010) Right hemisphere dominance during spatial selective attention and target detection occurs outside the dorsal frontoparietal network. J Neurosci 30:3640–3651

4. Isensee F, Kickingereder P, Wick W, et al (2017) Brain tumor segmentation and radiomics survival prediction: Contribution to the brats 2017 challenge. In: International MICCAI Brainlesion Workshop. Springer, pp 287–297

5. Luckett PH, Lee JJ, Park KY, et al (2022) Resting state network mapping in individuals using deep learning. Front Neurol 13:

6. Székely GJ, Rizzo ML, Bakirov NK (2007) Measuring and testing dependence by correlation of

distances. Ann Stat 35:2769–2794

7. Breiman L (2001) Random forests. Mach Learn 45:5–32

8. Fisher A, Rudin C, Dominici F (2019) All Models are Wrong, but Many are Useful: Learning a Variable’s Importance by Studying an Entire Class of Prediction Models Simultaneously. J Mach Learn Res 20:1–81
