# Supplementary material for: Predicting post-surgical functional status in high-grade glioma with resting state fMRI and machine learning
Source: J Neurooncol. 2024 May 24;169(1):175–85. doi: 10.1007/s11060-024-04715-1 (PMC11269343; doi:10.1007/s11060-024-04715-1)
Supplement: Supplementary file 2 — Supplementary Material 2 [file 11060_2024_4715_MOESM2_ESM.pdf]

	All Data	KPS<70	KPS≥70	p-value
N	102	21	81	
Survival(m) (Median±MAD)	16.1±13.5	12.1±8.8	18.3±14.2	.02
Age(y) at Diagnosis (Mean±STD)	56.1±14.6	65±12.2	53.8±14.4	.001
KPS-Surgery dates (Days, Median±MAD)	25±145	25±185	25±134	0.69
Sex (Male)	48.04%	42.9%	49.4%	0.59
Tumor hemisphere (%Right)	59.8%	61.9%	59.3%	0.98
STR	31.2%	28.6%	32.1%	0.76
NTR	17.7.3%	9.5%	19.8%	0.27
GTR	42.2%	52.4%	39.5%	0.28
LITT	8.8%	4.8%	9.9%	0.46
MGMT	43.1%	57.1%	39.5%	0.15
TERT	54.9%	66.7%	51.9%	0.22
PTEN	46.1%	57.1%	43.2%	0.25
EGFR	36.3%	33.3%	37.0%	0.75
IDH1	16.67%	4.8%	19.8%	0.1
Hx AUD	3.9%	0%	4.9%	0.3
Hx Tobacco	27.5%	19.1%	29.6%	0.33
Hx HTN	42.2%	71.4%	34.6%	0.002
Hx HLD	26.5%	47.6%	20.9%	0.01
Hx CKD	2%	4.8%	1.2%	0.3
Hx Cardiac	13.7%	19.1%	12.4%	0.43
Hx DVT/PE	2.9%	0%	3.7%	0.37
Hx Psychological	34.3%	47.6%	30.9%	0.15
Hx Visual Deficit	2%	4.7%	1.2%	0.3
Hx Stroke	2.9%	0%	3.7%	0.37
Hx Headaches	2.9%	4.7%	2.5%	0.58
Hx Weakness	2.9%	4.8%	2.5%	0.58
Hx Seizure	4.9%	0%	6.1%	0.24
Pw Aphasia	24.5%	14.3%	27.2%	0.22
Pw Weakness	34.3%	47.6%	30.9%	0.15
Pw Visual_Changes	16.7%	19.1%	16.1%	0.73
Pw Confusion	29.4%	33.3%	28.4%	0.66
Pw Headaches	42.2%	23.8%	46.9%	0.06
Pw Memory_Imp	12.8%	9.5%	13.6%	0.62
Pw Seizure	24.5%	57.1%	16.1%	<0.001
Pw Obesity	24.5%	28.6%	23.5%	0.63
Pw Diabetes	13.7%	23.8%	11.1%	0.13

Supplemental Table 1. Patient Demographics. Continuous data used the Kruskal-Wallis test; ratios used the chi-squared test. Hx = History of, Pw = presented with, AUD = alcohol use disorder, HTN = hypertension, HLD = hyperlipidemia, CKD = chronic kidney disease.
